# Directed funding to address under-provision of treatment for substance use disorders: a quantitative study

**DOI:** 10.1186/1748-5908-8-79

**Published:** 2013-07-18

**Authors:** Austin B Frakt, Jodie Trafton, Amy Wallace, Matthew Neuman, Steven Pizer

**Affiliations:** 1 Healthcare Financing & Economics, VA Boston Healthcare System and School of Medicine, Boston University, Boston, MA, USA; 2 Program Evaluation and Resource Center, VA Palo Alto Healthcare System, Menlo Park CA, USA; 3 Department of Psychiatry, Veterans Administration North Texas Health Care System, Bonham, TX, USA

**Keywords:** Substance use disorder, Veterans, Veterans Health Administration, Funding

## Abstract

**Background:**

Substance use disorders (SUDs) are a substantial problem in the United States (U.S.), affecting far more people than receive treatment. This is true broadly and within the U.S. military veteran population, which is our focus. To increase funding for treatment, the Veterans Health Administration (VA) has implemented several initiatives over the past decade to direct funds toward SUD treatment, supplementing the unrestricted funds VA medical centers receive. We study the ‘flypaper effect’ or the extent to which these directed funds have actually increased SUD treatment spending.

**Methods:**

The study sample included all VA facilities and used observational data spanning years 2002 to 2010. Data were analyzed with a fixed effects, ordinary least squares specification with monetized workload as the dependent variable and funding dedicated to SUD specialty clinics the key dependent variable, controlling for unrestricted funding.

**Results:**

We observed different effects of dedicated SUD specialty clinic funding over the period 2002 to 2008 versus 2009 to 2010. In the earlier period, there is no evidence of a significant portion of the dedicated funding sticking to its target. In the later period, a substantial proportion—38% in 2009 and 61% in 2010—of funding dedicated to SUD specialty clinics did translate into increased medical center spending for SUD treatment. In comparison, only five cents of every dollar of unrestricted funding is spent on SUD treatment.

**Conclusions:**

Relative to unrestricted funding, dedicated funding for SUD treatment was much more effective in increasing workload, but only in years 2009 and 2010. The differences in those years relative to prior ones may be due to the observed management focus on SUD and SUD-related treatment in the later years. If true, this suggests that in a centrally directed healthcare organization such as the VA, funding dedicated to a service is a necessary, but not sufficient condition for increasing resources expended for that service.

## Background

Substance use disorders (SUDs) are a substantial problem in the United States (U.S.), and yet treatment seems to be underprovided, despite its documented benefits relative to cost. As of 2010, 8.9% of Americans age twelve and older (23 million individuals) had an identified substance use problem. However, only 18% (four million) of them received treatment [1] despite the fact that treatment has been shown to be overwhelmingly cost-effective [2], as well as clinically effective [3]. Substance use is also a common problem among users of the Veterans Health Administration (VA), which provides care for U.S. military veterans through an integrated delivery system with salaried physicians [4]. Our study examined the effect on funding dedicated to VA SUD treatment on the amount of treatment actually delivered between 2002 and 2010. We consider the role of known changes in management that correlate with observed variation in the degree to which it did so.

SUD treatment is both effective for patients and has large, positive spillovers to society. The annual economic cost of excessive alcohol use alone has been estimated to be in the hundreds of billions of dollars [5]. In 1998, the economic cost of drug abuse in the U.S. was an estimated $143 billion, accounting for medical consequences, lost earnings and productivity, motor vehicle accidents, and crime [6]. Echoing earlier work [7,8], Parthasarathy *et al.* (2001) [9] found that SUD treatment is associated with a substantial offset effect, a subsequent reduction in emergency department and inpatient hospital use and costs. In a review of eleven studies, seven of which included sufficient information to calculate benefit-to-cost ratios, McCollister and French (2003) [10] found that benefits exceeded costs by a factor ranging from 1.33 to 23.33. Both Ettner *et al.* (2006) [2] and McCollister and French (2003) [10] attribute the majority of benefit to reductions in crime, a large minority to increases in income, and a smaller minority to avoided health spending. Basu *et al.* (2008) [11] found that outpatient methadone and residential SUD treatment is associated with a large reduction in armed robberies (0.4 per client per year), the value of which exceeds the cost of treatment by itself. Although most of the benefits of SUD treatment are social and economic, the health benefits may be significant enough to offset the costs, at least at the program level. For example, Wickizer *et al.* (2012) [12] studied the expansion of SUD treatment financed by the Washington State Medicaid program in the mid-2000s. They found that healthcare spending avoided by the state’s Medicaid program due to SUD treatment almost fully offset the cost of treatment to the program.

As these studies document, SUD treatment works, but its benefits are often diffuse, accruing in large part to society or broad levels of government and not the patient or the patient’s particular health services provider. Therefore, the benefits do not provide a strong incentive for managers of health systems to devote more resources to treatment. As Humphreys *et al.* (2011) [13] wrote, ‘If, for example, one is held responsible to keep a hospital budget in balance, spending scarce funds on SUD treatment does not become more attractive just because it saves money for the prison system.’ Attitudes and beliefs about SUD patients and treatments may also play a role in limiting funding and availability of treatment. For example, a report by the National Center on Addiction and Substance Abuse (2000) [14] found that physicians think other health conditions like hypertension, diabetes, and depression are far more treatable than substance use; over 40% of physicians have difficulty discussing alcohol or prescription drug abuse with patients; and the leading reason why physicians don’t discuss SUDs with patients is that they think ‘patients often lie.’ Given findings like these, it is not unreasonable to think that attitudinal factors might contribute toward less SUD treatment than would be optimal given the cost-benefit trade-off.

Consequently, because of its positive externalities, SUD treatment is underprovided relative to its broad benefits. Indeed, compared to the need for and benefit of treatment, funding for SUD treatment is modest [15], accounting for just 1.2% of all U.S. health spending in 2005. Growth in funding for SUD treatment has been at a rate far below that of total healthcare spending (4.8% versus 7.9% over 1986 to 2005), reaching $22 billion in 2005 [16].

A natural solution to the problem of underinvestment in and under-provision of SUD treatment is to place SUD treatment funding decisions in the hands of a more centralized authority that has responsibility for all or more of the areas it affects. If successful, centralized funding dedicated to SUD treatment would increase the resources devoted to it. This is precisely what block grants, like the Substance Abuse Prevention and Treatment Block Grants (SAPTBG) administered by the U.S. Substance Abuse and Mental Health Services Administration (SAMHSA), are designed to do. A similar directed funding effort, which we examine, exists within the VA.

Across many settings, economists have found that, relative to unrestricted resources, centrally administered, dedicated funds have a much larger effect on spending for the services to which they are targeted, including SUD treatment. Funds ‘sticking where they hit’ or where they are targeted is known as a ‘flypaper effect’ in the public finance economics literature [17]. However, the flypaper effect may vary in strength because some dedicated funds may not stick. It is important, therefore, for policymakers and SUD treatment advocates to measure how much sticks and understand the factors that cause it to do so. More specifically, the questions we address are: Are VA dedicated funds allocated to SUD treatment used for treatment in greater proportion than unrestricted funds? If so, to what extent? And what factors might explain any observed differences in the strength of flypaper effects over time and relative to non-VA settings? Though ours is the first study of the flypaper effect for VA SUD treatment, it has been examined by others outside the VA. Both Huber *et al.* (1994) [18] and Gamkhar and Sim (2001) [19] found that a one dollar increase in U.S. federal-to-state SUD treatment block grant funding led to an 80-cent increase in spending on alcoholism treatment. Jacobson and McGuire (1996) [20] and Ma *et al.* (2002) [21] found closer to a dollar-for-dollar relationship. Other than these studies, there have been no other investigations of the flypaper effect for SUD funding.

Recognizing the gap between SUD treatment need and capacity, in the period we study (2002 to 2010) the VA collected considerable data on SUD treatment programs. Over the same period, the VA initiated several programs motivated by a unified effort to direct funds toward SUD and broader mental health treatment. Under provisions of the Veterans Millennium Health Care and Benefits Act of 1999 (Pub. Law 106–117), Congress directed about $30 million between 2000 and 2002 to hiring additional VA SUD treatment staff^a^. Several years after the Millenium Act, the Veterans Health Administration Comprehensive Mental Health Strategic Plan, adopted in 2004, aimed to remove identified gaps in the VA’s provision of mental health services [22]. As part of the VA’s Mental Health Enhancement Initiative, which commenced in 2005, the department enhanced funding of mental health programs generally and SUD-specific treatment in particular. This was followed up in 2008 by the VA Mental Health and Other Care Improvement Act (Pub. Law 110–387) and the adoption of the VA Uniform Mental Health Services Handbook [23]. Though under various laws and initiatives, these efforts constituted a more-or-less consistent response to a perceived need to increase mental health and SUD spending and treatment within the VA.

In total, between 2002 and 2010, the VA directed about $152 million in centrally administered funds toward hiring additional SUD treatment staff. The degree to which these funds were used for their intended purpose has not been broadly investigated. Using descriptive methods very different from the multivariate approach we take, the U.S. Government Accountability Office (2006) [24] examined the use of 2005 and 2006 funding associated with the 2004 VA Mental Health Strategic Plan, finding that some of the funding was not applied to its targeted use. The level and geographic targeting (specific VA facilities) of SUD-directed funding varied over the period of study, providing an opportunity to more thoroughly assess the effectiveness of increasing resources dedicated to treatment within the VA. Our work is the first such comprehensive assessment.

## Methods

The study sample included all VA facilities and used observational data spanning years 2002 to 2010. We follow the approach of Jacobson and McGuire (1996) [20] and estimate the effects of dedicated funding a with fixed effects^b^, ordinary least squares (OLS) regression model with total resources expended on the target service as the dependent variable and dedicated funding amounts as the key independent variable, controlling for variations in the broader budget. This is an important control because an increase in total resources available to local policymakers ought to lead to higher spending on everything, including the target service. Fixed effects control for permanent differences between localities (VA medical centers in our application). Additionally, we have nine years of data, so we include year effects and interact them with dedicated funding amounts to assess whether dedicated funds had different effects through time. We used a Newey-West (1987) [25] variance estimator to correct for autocorrelation and possible heteroskedasticity. The equation below specifies the model, where the unit of analysis is the medical center-year:

$$\begin{array}{l} {\left(\mathrm{monetized}\;\mathrm{workload}\right)}_{m,y}\\ \;=\left(\mathrm{unrestricted}\;\mathrm{medical}\;\mathrm{center}\;\mathrm{budget}\;\mathrm{allocation}\right)\\ \;+\;\beta_{y}{\left(\mathrm{SUD}\;\mathrm{specialty}\;\mathrm{clinic}\;\mathrm{dedicated}\;\mathrm{funding}\right)}_{m,y}{+\gamma }_{m}{+\delta }_{y}{+\epsilon }_{m,y} \end{array}$$

The variables in the equation—monetized workload, unrestricted medical center budget allocation, SUD specialty clinic dedicated funding—are described in the subsections that follow. In the equation, *m* indexes medical centers, *y* years. The parameters α to δ are estimated by ordinary least squares (OLS) using Stata 10 [26]. Note that α is fixed across years and medical centers, β is year varying, γ are medical center fixed effects, δ are year fixed effects, and ϵ is the error term. To estimate the model we constructed an analytic file consisting of 1,142 medical center-year observations, spanning the years 2002 to 2010. The file includes the variables shown in Table 1. The construction of each of those variables, and the data used to do so, is described below.

**Table 1:** Variable definitions and univariate statistics

**Variable**	**Mean (SD)**	**[Min–Max]**
Dependent variable		
Monetized SUD clinic workload	$2,568,794 ($2,243,658)	[$487 – $12,500,000]
Independent variables^(a)^		
Unrestricted budget allocation	22,300,000 (17,500,000)	[936,168 – 114,000,000]
SUD clinics funding x 2002	7,708 (50,316)	[0 – 495,300]
SUD clinics funding x 2005	7,214 (39,977)	[0 – 344,759]
SUD clinics funding x 2006	24,614 (101,506)	[0 – 976,527]
SUD clinics funding x 2007	36,362 (145,709)	[0 – 1,287,950]
SUD clinics funding x 2008	25,025 (122,373)	[0 – 1,396,928]
SUD clinics funding x 2009	16,493 (95,028)	[0 – 1,096,928]
SUD clinics funding x 2010	16,493 (95,028)	[0 – 1,096,928]

(a) All years are binary variables, 1 for the year indicated, 0 otherwise.

N=1,142 year-station observations. Year and station fixed effects not shown.

Source: Authors’ tabulation of study data.

### Monetized workload

The dependent variable, monetized workload, is the product of the number of visits to the VA medical center’s SUD specialty clinics within the year made by VA patients with SUD diagnoses and an estimate of the cost of such a visit. The VA Office of Mental Health Operations Program Evaluation and Resource Center (PERC) analyzes SUD workload data, and from it we computed the number of visits made to SUD specialty clinics by patients with a principal diagnosis of a SUD. Our confidence in the reliability of these workload data is based on the work by Harris *et al.* (2010) [27], which found that the vast majority of patients with these administrative indications of specialty SUD treatment did in fact receive SUD care.

To monetize workload, we used a national average cost per visit derived from estimates of VA staff cost per visit, computed from VA staffing levels, wage, fringe, and indirect cost data from VA administrative sources. We computed a different national average cost per visit separately for each year as follows. First, for the denominator (visits), we summed our workload measure across medical centers within year.

The cost per visit numerator (SUD specialty clinic cost) was computed based on VA SUD specialty clinic treatment staff, weighted by salary, fringe, and indirect costs. We obtained VA SUD specialty treatment staffing data from the Drug and Alcohol Program Survey (DAPS), conducted in 2000, 2003, 2006, 2008, and 2010 by PERC [28-32]. For each medical center, these data include full-time equivalents (FTEs) for 15 types of staff (psychiatrist, physician, psychologist, physician assistant/nurse practitioner, RN/clinical nurse specialist, LP nurse/LV nurse, nursing assistant, social worker, addiction therapist/counselor (non-MSW), psychology/social work/rehabilitation tech or aide, pharmacist, recreational therapist, vocational rehabilitation specialist, secretary/administrative assistant/clerk, other). We aggregated these 15 types into four (medical management, advanced degree counselors, non-advanced degree counselors, support staff) and interpolated to produce national total counts of FTEs by these four staff types for all years 2002 to 2010, inclusive.

We obtained medical center and staff-type level total salary cost and hours data from the VA’s Account Level Budgeter Cost Center file, a VA accounting file validated for this purpose [33]. With these, we computed the average annual salary for each of the four aggregated staff types (medical management, advanced degree counselors, non-advanced degree counselors, support staff). We monetized the SUD treatment staff FTE counts by multiplying by these constructed average salaries and summing across staff type. We then scaled the resulting staff costs up for fringe and overhead using values from the Alcohol and Drug Services Study, a nationally representative study that collected the cost of providing care at substance abuse treatment facilities [34]^c^.

### Independent variables

With the exception of 2003 and 2004, between 2002 and 2010 (inclusive), medical centers received SUD treatment-dedicated funds. PERC tracked the precise amounts of dedicated SUD treatment funding allocated in each of those years. These year-specific SUD specialty dedicated funding variables are our key independent variables. (See the ‘SUD clinics funding’ variables in Table 1).

Because they also influence the level of resources devoted to SUD treatment, it is important to control for unrestricted funds. In the VA, unrestricted funds take the form of an allocation by Congress that is first distributed by formula to the 21 regional Veterans Integrated Service Networks (VISNs). VISN directors then divide their allocation among medical centers within each of their regions in ways that potentially depend on needs and priorities of the VISN and medical centers. Consequently, medical centers’ commitment to or ambition for SUD treatment can be a causal factor in the amount of unrestricted funds they receive. Likewise, unrestricted funds are a causal factor in amount of SUD treatment provided. In other words, unrestricted funds and funding allocated to SUD treatment are jointly determined. Because our interest is in the degree to which unrestricted funds influence SUD treatment workload, we use an approach developed by Pizer *et al.* (2004) [35] to extract a measure of unrestricted funds that cannot be caused by same-year changes in medical center commitment to SUD treatment. The method is based on assigning VISN-level funds to medical centers using the prior year’s distribution. See Pizer *et al.* (2004) [35] for details.

## Results

**Figure 1:**
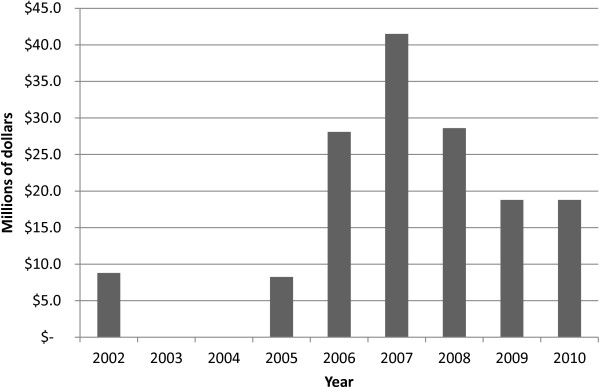
**Dedicated SUD specialty clinic funding, by year.** Source: Authors’ calculations using study data.

Table 1 provides descriptive statistics of the variables used in our study, based on the year-medical center file described above. All figures are in nominal dollars. The dependent variable is monetized SUD clinic workload. The independent variables are the medical centers’ unrestricted budget allocations and the dedicated funds for SUD by year. Notice that there was no dedicated SUD clinic funding in years 2003 or 2004. There was considerable variation in the level of dedicated funds by medical center, as reflected in the large standard deviations and min–max range of the dedicated funding variables. Figure 1 shows the total dedicated SUD clinic funding by year. Funding level peaked in 2007.

In Table 2, we provide OLS-estimated coefficients, standard errors, and two measures of statistical significance (one for the coefficient relative to zero and one for the coefficient relative to that for the unrestricted budget allocation). The unrestricted budget allocation has a positive and highly significant effect, as one would expect. More unrestricted funding is associated with more resources devoted to SUD clinics, at a rate of about five cents on the dollar. Most coefficients for dedicated SUD clinic funding are not statistically significant relative to zero or relative to the unrestricted budget allocation coefficient. The exceptions are in years 2009 and 2010. In those years, funding dedicated to SUD clinics was statistically significant and positively associated with monetized workload in SUD clinics. In 2009, 38 cents of each dollar dedicated to SUD clinics translated into additional workload. In 2010, 61 cents did so. Both are substantially higher than the five cents of each dollar of unrestricted budget allocation that is used for SUD treatment.

**Table 2:** Monetized workload regression

**Variable**	**Coefficient ** **(std. err.)**	**P-value relative to 0**	**P-value relativeto coefficient on unrestricted budget allocation**
Unrestricted budget allocation	0.049 (0.0048)	0.00	-
SUD clinics funding x 2002	−0.061 (0.43)	0.89	0.80
SUD clinics funding x 2005	−0.19 (0.41)	0.64	0.55
SUD clinics funding x 2006	0.089 (0.22)	0.69	0.86
SUD clinics funding x 2007	0.13 (0.14)	0.34	0.55
SUD clinics funding x 2008	0.23 (0.13)	0.081	0.17
SUD clinics funding x 2009	0.38 (0.19)	0.05	0.09
SUD clinics funding x 2010	0.61 (0.24)	0.01	0.02
	R^2^ = 0.52		

N=1,142 year-station observations. Year and station fixed effects not shown.

**Figure 2:**
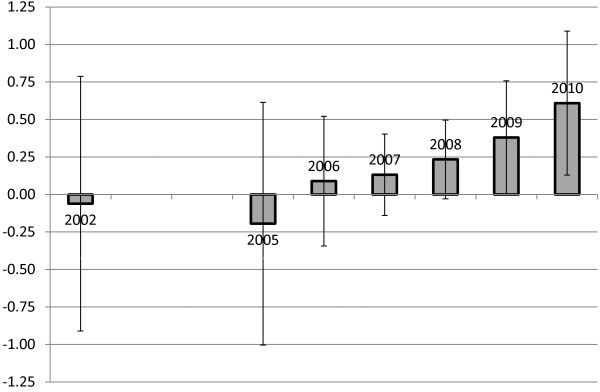
SUD clinic funding coefficients and 95% confidence intervals.

Figure 2 illustrates the SUD clinics funding coefficients shown in Table 2, as well as their 95% confidence intervals. Over time, a greater proportion of dedicated funding was applied to SUD treatment.

## Discussion

Providing dedicated funding for SUD treatment from a central authority potentially addresses one problem and raises another. It potentially addresses the fact that SUD treatment has positive externalities from which local healthcare managers do not benefit (and of which they may not even be aware). Consequently, local decision making about the appropriate level of resources to devote to SUD treatment can lead to under-provision of care. Though dedicated funding can increase spending on SUD treatment, it only does so to the extent that it does not offset unrestricted funds. The key question about dedicated funding schemes is: how much dedicated funding sticks where it hits, both in absolute terms and relative to unrestricted funding? Or, how big is the ‘flypaper’ effect?

We addressed this question in the context of SUD treatment within the VA and found different effects over the period 2002 to 2008 versus 2009 to 2010. In the earlier period, there is no evidence of a significant portion of the dedicated funding sticking to its target, SUD specialty clinics. In the later period, a substantial proportion—38% in 2009 and 61% in 2010—of funding dedicated to SUD specialty clinics did translate into increased medical center spending for SUD treatment. Relative to a dollar of unrestricted funding, only five cents of which is spent on SUD specialty treatment, according to our estimates, dedicated funding has large flypaper effects. But why are such effects absent prior to 2009?

One explanation is that in 2002 to 2008 dedicated funding targeted treatment in SUD specialty clinics only, whereas by 2009 to 2010 there was an effort to increase SUD treatment provided in other mental health settings. This coincides with a longer effort to bolster VA mental health, including a focus by VA Secretary Shinseki on combating homelessness [36], a problem closely tied to substance use [37]. The Veterans Health Administration Comprehensive Mental Health Strategic Plan, adopted in 2004, aimed to remove identified gaps in the VA’s provision of mental health services [22]. As part of the VA’s 2005 Mental Health Enhancement Initiative, The VA enhanced funding of mental health programs generally. This was followed up in 2008 by the VA Mental Health and Other Care Improvement Act (Pub. Law 110–387) and the adoption of the VA Uniform Mental Health Services Handbook [23]. It is possible that in the early years of our study and before mental health clinics received all of these additional supports, funds dedicated to SUD specialty clinics were redirected to functions within mental health departments. Humphreys *et al.* (1997) [38] noted diversion of SUD treatment funds to other uses within VA medical centers has been observed. In 2009 and 2010, with separate funds dedicated to SUD treatment outside specialty clinics and within mental health, perhaps there was less of a need for medical centers to divert funds dedicated to SUD specialty clinics. Also, the VA Uniform Mental Health Services Handbook, released in 2009, explicitly required and monitored availability of intensive outpatient and residential SUD treatment, including opioid agonist treatment, and these explicit policy requirements may have increased prioritization of SUD programs in funding decisions.

Another possibility is that a longer duration focus on building SUD treatment capacity also increases stakeholder investment. Local stakeholders may become more numerous and experienced, which could translate into better advocacy for resources. If this is the case, then one should expect flypaper effects to grow over time, which is what we found. We also considered the possibility that monitoring of use of dedicated funds changed over time. However, discussion with PERC staff did not reveal obvious changes that seemed to correlate with the timing of our findings.

Our work raises another interesting question: why do we find flypaper effects substantially below those documented for state agencies? Huber *et al.* (1994) [18], Gamkhar and Sim (2001) [19], Jacobson and McGuire (1996) [20], and Ma *et al.* (2002) [21] all found that a dollar of SAPTBG funding led to at least 80 cents of additional spending for SUD treatment, yet we find, at most, a 61 cents on the dollar effect. Methodological differences cannot be discounted. In addition, Keith Humphreys, former Senior Policy Advisor at the White House Office of National Drug Control Policy, offered two other, possible explanations for the difference (personal communication). First, in some cases, SAPTBG funding is often directed toward mono-mission agencies. As such, there is no scope for diversion toward other purposes within the agency. In contrast, VA dedicated funding is always directed toward multi-mission medical centers. That at least provides the necessary conditions for diversion and also makes use of funds harder to monitor. Second, in many states (Arkansas being one) agencies that receive SAPTBG funds are not substantially funded in any other way. Consequently, there is no unrestricted funding to redirect that could offset SAPTBG funds.

The VA can’t be made to resemble state agencies that receive SAPTBG funds. It is a multi-mission agency tasked with providing comprehensive healthcare rather than just SUD treatment. Cordoning off SUD funding, as in state agencies, would discourage integration of SUD treatment with general medical care and would encourage the continued stigma of SUDs. Thus, eliminating the fungibility of VA centralized funds as in state agencies is counter to aspects of the VA mission. What, then, could the VA do to further increase the flypaper effect for centralized SUD funding initiatives? First, efforts to reduce conflict between local and central priorities should improve the ‘stick’ of centralized funding. This might be accomplished by ensuring that responsibility for implementation of centralized initiatives is held by those making funding decisions. Second, implementation of centralized initiatives could be monitored within the overall context of SUD staffing and programming, rather than separately.

We note that based on experience with implementation of these initiatives, VA has changed practices along these lines. In 2011, VA realigned its central office to place responsibility for implementation of centralized funding initiatives under the same leadership responsible for management of the local unrestricted medical center budgets. Additionally in 2011, to support adoption of the Uniform Mental Health Services Handbook, VA implemented a monitoring and quality improvement program including an intranet-based Mental Health Information System with over 200 measures indexing the level of delivery of services outlined in the Handbook, and a technical assistance and site visit program that helps facilities identify and correct deficiencies in mental health services delivery. Both of these modifications increase the accountability of the local leadership who manage the unrestricted budgets for SUD service delivery and centralized initiative implementation. The VA has also expanded its monitoring of centralized initiatives to track not only whether centralized initiative funding was spent on its designated purpose, but also overall staffing levels, filling of vacant staffing positions, and workload across SUD and other mental health treatment domains to ensure that initiative-driven expansions are not at the expense of related services.

There are a few, final points we wish to emphasize. First, one limitation of our work is that the dedicated funding figures available for this study are the amounts that were sent to medical centers. Though it is known that some medical centers returned some of the funding because they were unable to use it [24], we have no systematic, comprehensive data on where and to what extent this occurred. Related, our work suggests that some of the funding intended for specialty SUD treatment was used for other purposes. Likely much of it was retained for broader mental healthcare delivery and/or for SUD treatment in non-specialty settings. At this time, the data resources within the VA are insufficiently mature to conduct an analysis of broader mental health or SUD treatment in non-specialty settings similar to that presented above for specialty SUD treatment. This is an area worthy of future investigation.

Last, but certainly not least, we do not mean to imply that a flypaper effect that is below one is necessarily bad. In fact, as we pointed out, only five cents of every dollar of unrestricted funding is spent on SUD treatment. Relative to this, the degree to which funds dedicated to SUD treatment hit their target in 2009 and 2010 was very large. Still, it never reached close to unity, suggesting that some of the funds were used for other purposes. Medical center directors who redirect some SUD funding to other purposes likely have good reason to do so. The care those redirected funds support benefits veterans, and may even be used for SUD treatment in non-specialty settings. Though we can only speculate why (as we did above), the flypaper effect of VA dedicated SUD funding has gone up over time. To the extent that central administrators aim to increase resources for SUD treatment, that is probably welcome news to them.

## Conclusions

Relative to unrestricted funding, dedicated funding for SUD treatment was much more effective in increasing workload, but only in years 2009 and 2010. The differences in those years relative to prior ones may be due to an observed management focus on SUD and SUD-related treatment in the later years. If true, this suggests that in a centrally directed healthcare organization such as the VA, funding dedicated to a service is a necessary, but not sufficient condition for increasing resources expended for that service.

## Endnotes

^a^Dollar figures in this paragraph and next were calculated by the authors based on data provided by the VA Office of Mental Health Operations Program Evaluation and Resource Center. Those data are described in the Methods and Data section.

^b^We conducted a Hausman specification test of fixed vs. random effects, and that it overwhelmingly rejected the hypothesis that the difference in fixed and random effects coefficients was not systematic (P-value < 0.0001). Hence, a random effects specification was rejected.

^c^We used one, national rate rather than station-specific rates from the VA because Barnett and Berger (2003) [39] found that VA station-specific overhead rates vary widely ‘due to the differences in the definition of direct costs used by different facilities.’ The inpatient treatment overhead rate derived from ADSS is almost the same as that from the VA-specific one, 0.63 from ADSS vs. 0.57 derived from Barnett and Berger (2003) [39].

## Competing interests

The authors declare that they have no competing interests.

## Authors' contributions

ABF carried out the analysis and wrote the initial draft of the paper. JT provided institutional and editorial input, and advised on appropriate use of data. AW provided institutional, clinical, and editorial input. SDP lead the study design and edited the paper. All authors read and approved the final manuscript.
